# Acute Phosphate Restriction Leads to Impaired Fracture Healing and Resistance to BMP-2

**DOI:** 10.1359/jbmr.091021

**Published:** 2009-10-17

**Authors:** Nathan A Wigner, Hilary F Luderer, Megan K Cox, Karen Sooy, Louis C Gerstenfeld, Marie B Demay

**Affiliations:** 1 Orthopaedic Research Laboratory, Department of Orthopaedic Surgery, Boston University Medical CenterBoston, MA, USA; 2 Endocrine Unit, Massachusetts General Hospital and Harvard Medical SchoolBoston MA, USA

**Keywords:** rodent, fracture, BMP, endochondral, intramembranous, hypophosphatemia

## Abstract

Hypophosphatemia leads to rickets and osteomalacia, the latter of which results in decreased biomechanical integrity of bones, accompanied by poor fracture healing. Impaired phosphate-dependent apoptosis of hypertrophic chondrocytes is the molecular basis for rickets. However, the underlying pathophysiology of impaired fracture healing has not been characterized previously. To address the role of phosphate in fracture repair, mice were placed on a phosphate-restricted diet 2 days prior to or 3 days after induction of a mid-diaphyseal femoral fracture to assess the effects of phosphate deficiency on the initial recruitment of mesenchymal stem cells and their subsequent differentiation. Histologic and micro-computed tomographic (µCT) analyses demonstrated that both phosphate restriction models dramatically impaired fracture healing primarily owing to a defect in differentiation along the chondrogenic lineage. Based on *Sox9* and *Sox5* mRNA levels, neither the initial recruitment of cells to the callus nor their lineage commitment was effected by hypophosphatemia. However, differentiation of these cells was impaired in association with impaired bone morphogenetic protein (BMP) signaling. In vivo ectopic bone-formation assays and in vitro investigations in ST2 stromal cells confirmed that phosphate restriction leads to BMP-2 resistance. Marrow ablation studies demonstrate that hypophosphatemia has different effects on injury-induced intramembranous bone formation compared with endochondral bone formation. Thus phosphate plays an important role in the skeleton that extends beyond mineralized matrix formation and growth plate maturation and is critical for endochondral bone repair. © 2010 American Society for Bone and Mineral Research.

## Introduction

The skeleton is an organ that is subject to constant remodeling and repair. Fracture initiates a series of molecular and cellular events that recapitulate embryonic endochondral bone formation. Upon fracture of long bones, a hematoma is formed, followed by an inflammatory phase characterized by expression of numerous cytokines. In addition to a periosteal reaction adjacent to the fracture site, there is recruitment of mesenchymal cells into the area of the fracture. These cells then differentiate into cartilage under the influence of bone morphogenetic proteins (BMPs). The maturing cartilaginous callus is invaded by vasculature and replaced by bone. Remodeling of the callus leads to restoration of the anatomic and biomechanical properties of the original skeletal element.(1)

A number of factors have been shown to influence fracture repair, and analyses of the molecular basis for these abnormalities has led to a greater understanding of this process.(2–7) Diabetes mellitus leads to impaired fracture healing owing to premature resorption of the cartilaginous callus.(6) Prostaglandins also have been shown to play an important role in fracture repair,(5) a seminal finding that has led to the avoidance of nonsteroidal anti-inflammatory medications, which inhibit the rate-limiting step in prostaglandin production, in patients with fractures. At 14 days, the calluses of *Cox2* null mice exhibit a decrease in the osseous content, accompanied by a persistence of mesenchymal cells,(5) impaired expression of BMP-2 and BMP-7.(8,9) and decreased angiogenesis.(10)

Hypophosphatemia is a feature of several congenital rachitic disorders.(11–13) Investigations in murine models of X-linked hypophosphatemia and hereditary vitamin D–resistant rickets demonstrate that rachitic expansion of the hypertrophic chondrocyte layer in the growth plate is a consequence of impaired apoptosis of these cells.(14) Molecular analyses reveal that increases in extracellular phosphate activate the mitochondrial apoptotic pathway in hypertrophic chondrocytes, a process that is required for normal endochondral bone formation. Thus, while phosphate is required for mineralized matrix formation, it also plays a regulatory role in terminal differentiation of hypertrophic chondrocytes.

In addition to rickets, congenital and acquired hypophosphatemic states are associated with impaired fracture healing.(11–13,15) However, it is unclear what specific role phosphate plays in this process. These disorders are associated with osteomalacia owing to impaired matrix mineralization, which may itself have an important effect on fracture healing. Furthermore, the hypophosphatemia associated with oncogenic osteomalacia and X-linked hypophosphatemia is accompanied by a decrease in 1,25-dihydroxyvitamin D and an increase in fibroblast growth factor 23 (FGF-23) levels,(16) which may contribute to impaired fracture healing. Similarly, impaired vitamin D action may play a role in the abnormal fracture healing observed with vitamin D deficiency. Because neither the cellular nor the molecular basis for impaired fracture repair in these conditions has been examined, we undertook investigations to determine if acute phosphate restriction alters fracture healing and to identify the mechanism by which hypophosphatemia leads to impaired skeletal repair.

## Materials and Methods

### Animal maintenance

Animal studies were approved by an institutional animal care committee. Male C57Bl/6J (B6) mice were purchased from Jackson Laboratories (Bar Harbor, ME, USA) and fed standard chow (Teklad 2018, Madison, WI, USA; 0.65% phosphorus) or a phosphate-restricted diet (PRD; 0.06% P_i_). Under anesthesia, a closed mid-diaphyseal fracture of the left femur was generated by controlled blunt trauma using a scaled-down modification of the apparatus described by Bonnarens and Einhorn.(17) Ectopic bone-formation assays were performed under anesthesia by implanting a Helistat sponge containing 5 µg recombinant human (rh) BMP-2 (a gift from Wyeth, Andover, MA, USA) or saline, following which the mice were fed the control or phosphate-restricted diet. Marrow ablation studies(3) were performed after 2 days of phosphate restriction in 6-week-old C57Bl/6J mice. All animals were euthanized by CO_2_ asphyxiation prior to harvesting tissue.

### Histology

Specimens were fixed, decalcified, and processed for 5 µm paraffin sections and stained with hematoxylin and eosin or saffranin O and fast green. Histomorphometry was performed using a Nikon E800 microscope and Osteomeasure software (Osteometrics, Inc., Atlanta, GA, USA). Proliferation was evaluated using a Zymed PCNA staining kit (Invitrogen, Carlsbad, CA, USA). The percent of proliferating cell nuclear antigen (PCNA)-positive stromal cells 7 days after fracture was determined by evaluating 500 stromal cells on three sections obtained from three fractures for each condition. Evaluation of nuclear phospho-Smad was performed using the TSA-biotin kit and anti-phospho-Smad (Upstate Biotechnology, Lake Placid, NY, USA). In situ hybridization was performed using ^35^S-UTP-labeled antisense riboprobes as reported previously.(18,19)

### Quantitative micro-computed tomography (µCT)

Scans were performed using a Scanco µCT 40 system (Scanco Medical, Basserdorf, Switzerland) at 12 µm voxel resolution with 200 ms integration time under conditions of 55*E* (KVp) and 145*I* (µA). Reference lines were adjusted manually on individual bones to include the entire callus area. Transverse images were reconstructed digitally to generate a 3D image of the callus. Analyses were carried out using Scanco Medical software. A global threshold algorithm was used to apply a fixed constant threshold to all specimens. A constrained 3D Gaussian filter (σ = 0.8, filter support = 1 voxel) was used to partially suppress image noise.

### mRNA analyses

The callus was isolated from adjacent tissue, and total RNA was extracted using Trizol (Invitrogen) and purified on RNeasy minicolumns (Qiagen, Valencia, CA, USA). Quantitative real-time polymerase chain reaction (RT-PCR) was performed using Taqman MGB expression assays (Applied Biosystems, Foster City, CA, USA) on an ABI 7700 Sequence Detector (Applied Biosystems). All samples were run in triplicate and normalized to the average threshold value of triplicate determinations of β-actin in the same sample. The fold change in mRNA was normalized to the samples from unfractured control bone (C57Bl/6J day 0).

### Biochemical analyses

Serum phosphate and calcium levels were measured using the Phosphorus Liqui-UV Kit and Calcium CPC LiquiColor Test Kit (Stanbio Laboratory, Boerne, TX, USA).

### Cell culture and immunocytochemistry

ST2 stromal cells were cultured in Roswell Park Memorial Institute Media (RPMI) (5.6 mM phosphate), 10% fetal bovine serum (FBS), and 1% penicillin/streptomycin. To evaluate the effect of hypophosphatemia on BMP-2-induced nuclear phospho-Smad, cells were plated in DMEM (1 mM phosphate) 10% FBS, and 1% penicillin/streptomycin 5 days prior to BMP-2 treatment or kept in basal medium. Twenty-four hours prior to BMP-2 treatment, 1% heat-inactivated FBS was substituted for the 10% FBS. Cells were treated with 200 ng/mL of BMP-2 for 90 minutes. Immunocytochemistry was performed using anti-pSmad (Upstate Biotechnology) and detected with a streptavidin–Texas red–conjugated goat anti-rabbit antibody (Perkin Elmer, Waltham, MA, USA). Slides were cover-slipped with 4',6-Diamidino-phenylindole (DAPI)-containing mounting medium to permit identification of cell nuclei.

### Western blot analysis

Membranes were blocked in 5% milk/Tris-buffered saline/Tween-20 (TBST) (Upstate Biotechnology) and incubated with anti-pSmad, and immunodetection was performed with an anti-rabbit horseradish peroxidase (HRP)–conjugated secondary antibody. Blots were reprobed for actin as a loading control. Protein expression was visualized using a Western Lightening Plus-ECL detection kit (Perkin Elmer).

### Statistical analysis

For µCT analyses, a two-factor ANOVA using the effects of time, treatment, and the interaction between time and treatment was used. A Tukey post hoc least significant difference (LSD) test was used to identify significant difference between the means. For RT-PCR analyses, the difference between the mean values of the mRNA expression and control at each time point was analyzed by Student's *t* test; p < .05 was considered significant.

## Results

To determine whether phosphate plays a role in mesenchymal cell and chondrocyte differentiation during fracture repair, the effect of a phosphate-restricted diet (PRD) was assessed in a murine model of fracture healing. In this model, the process of fracture healing consists of four temporally defined phases of repair(1): an early phase characterized by an inflammatory response with recruitment of mesenchymal cells (days 1 to 4), an early intermediate phase in which the phenotype associated with differentiation of these cells into chondrocytes predominates (days 4 to 10), a late intermediate stage marked by endochondral resorption and primary bone formation (days 10 to 16), and the final stage in which secondary bone formation occurs (days 16 to 21).

**Fig. 1:**
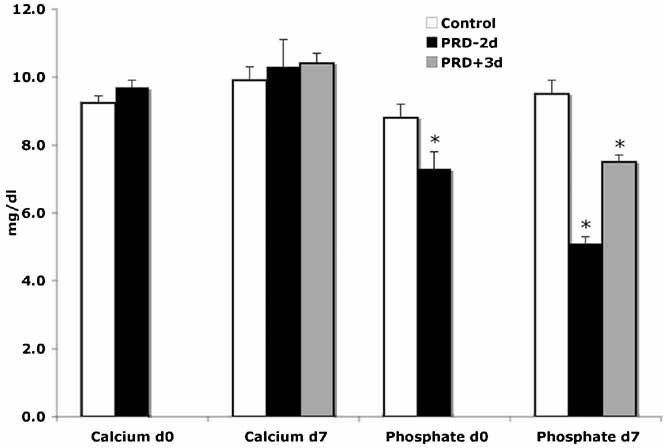
Serum mineral ion levels. Calcium and phosphate levels of mice fed normal chow (control, *white bars*), mice placed on the PRD 2 days prior to fracture (PRD–2d, *black bars*), and mice placed on the PRD 3 days after fracture (PRD+3d, *gray bars*) were measured the day of fracture (d0) and 1 week after fracture (d7). Data represent the mean and SEM of samples obtained from 7 to 10 mice for each condition. (^*^p < .05 versus controls.).

Mice were placed on the PRD 2 days before induction of a femoral fracture (PRD–2d) to evaluate whether hypophosphatemia impairs the recruitment of mesenchymal cells to the fracture site. A second group of mice was placed on the PRD 3 days after fracture (PRD+3d) to determine whether hypophosphatemia affects the differentiation of cells recruited to the callus site. The latter model was used to circumvent the effects of hypophosphatemia on the initial inflammatory and mesenchymal cell recruitment stages of fracture healing. Biochemical determinations were performed to evaluate the onset and degree of hypophosphatemia in this model. As shown in Fig. 1, the serum phosphate level was significantly decreased at the time of fracture in the mice subjected to the PRD 2 days prior to fracture (PRD–2d, day 0).

Initial analysis of callus formation was performed using µCT at 14 and 21 days after fracture, a time when evaluation of callus size and mineralization is amenable to this technique. 3D µCT reconstruction revealed that hypophosphatemia decreased the overall callus size and mineralization in both PRD groups 14 and 21 days after fracture (2*A*). Regardless of dietary regimen, peak total callus volume was achieved by day 14. Both phosphate-restriction protocols led to a reduction in total callus volume and cross-sectional area (see 2*B, C*). Mineralized tissue volume and callus mineral content (see 2*D, E*) also were significantly reduced, whereas a modest decrease in callus mineral density was observed (see 2*F*) in the mice on the PRD. A reduction in the ratio of mineralized tissue volume to total callus volume was observed in the mice on the PRD (see 2*G*).

**Fig. 2:**
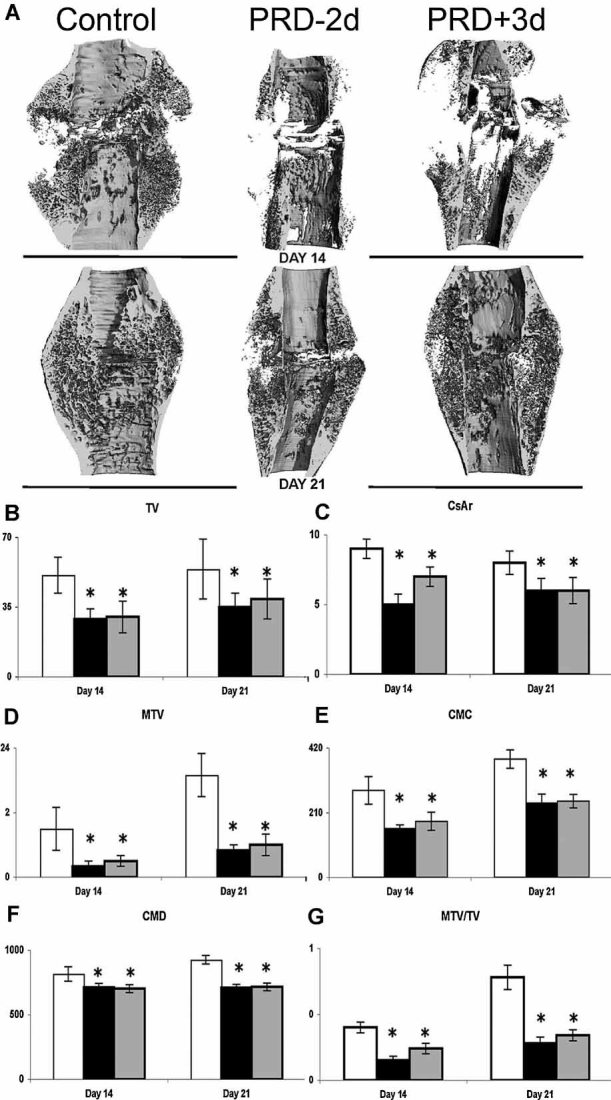
µCT analyses of callus formation. A. 3D µCT reconstructed images of fracture calluses of mice on the control and PRD diets at 14 days (*upper panel*) and 21 days (*lower panel*) after fracture. Data are representative of images obtained from 5 mice for each experimental condition. Quantitative µCT analyses of fracture healing 14 and 21 days after: (*B*) Total callus volume (TV). (*C*) Average callus cross-sectional area (CsAr). (*D*) Mineralized tissue volume (MTV). (*E*) Callus mineral content (CMC). (*F*) Callus mineral density (CMD). (*G*) Relative mineralized tissue volume (MTV/TV). *White bars*: Mice fed normal chow. *Black bars*: Mice placed on the PRD 2 days prior to fracture (PRD–2d). *Gray bars*: Mice placed on the PRD 3 days after fracture (PRD+3d). Data represent the mean and SD of values obtained from eight representative images of eight independent calluses that were used per treatment group and per time-point. ^*^p < .005 versus controls.

**Fig. 3:**
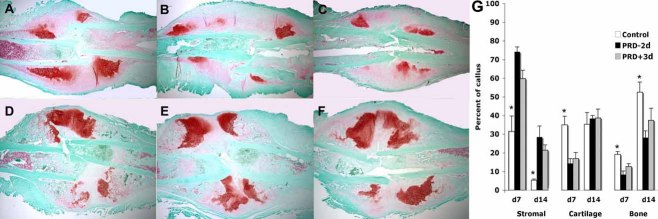
Callus histology and histomorphometry. Representative sections of callus formation 7 (*upper panels*) and 14 (*lower panels*) days after fracture in mice fed the control diet (*A*, *D*) or the phosphate-restricted diet 2 days before (*B*, *E*) or 3 days after (*C*, *F*) fracture. Cartilage stains red with saffranin O and stromal cells stain pink, whereas bone stains green with fast green. (*G*) Histomorphometry. Percentages of the callus area occupied by stromal cells, cartilage, and bone are indicated. Data represent the mean value obtained from analyses of five representative sections from each of four calluses per time point. Significant differences (*p* < .05) are present between the experiental and control groups, with the exception of cartilage content at day 14.

To examine the effect of phosphate restriction on the recruitment and differentiation of cells in the callus, histologic analyses were performed 1 and 2 weeks after fracture. One week after fracture, the callus of the mice subjected to the PRD either 2 days before or 3 days after fracture was composed largely of stromal cells, with very little bone or cartilage (Fig. 3). In contrast, the calluses of mice on the control diet were composed of stromal cells and cartilage, as well as having a minor osseous component. At 2 weeks after fracture, all three groups demonstrated differentiation of cells toward a more mature phenotype, but the stromal cell compartment remained strikingly more prominent in the mice on the PRD, accompanied by a significantly smaller osseous compartment (see Fig. 3). Quantitative histomorphometric analyses of callus tissue composition confirmed the predominance of stromal cells in the callus of both groups of mice on the PRD at 7 days after fracture (see 3*G*). This was accompanied by a marked reduction in cartilage and bone relative to the callus of mice on the control diet. By day 14, the control callus exhibited a significant predominance of bone with a very minor stromal component. However, at this time point, the bone content of the calluses of the PRD groups was not significantly different from that of either their cartilage or stromal components. This difference in cellular composition largely was responsible for the decrease in callus volume of the mice on the PRD. Owing to the presence of a significant amount of matrix in the cartilaginous compartment, the cellular density of this compartment was one-third that of the stromal compartment. A smaller contribution to reduced callus volume was attributable to a modest decrease in stromal cell proliferation of (4.0 ± 0.5% and 3.7 ± 0.7% PCNA-positive stromal cells in the PRD–2d and PRD+3d groups versus 5.4 ± 0.1% in the control calluses, *p* = .04).

**Fig. 4:**
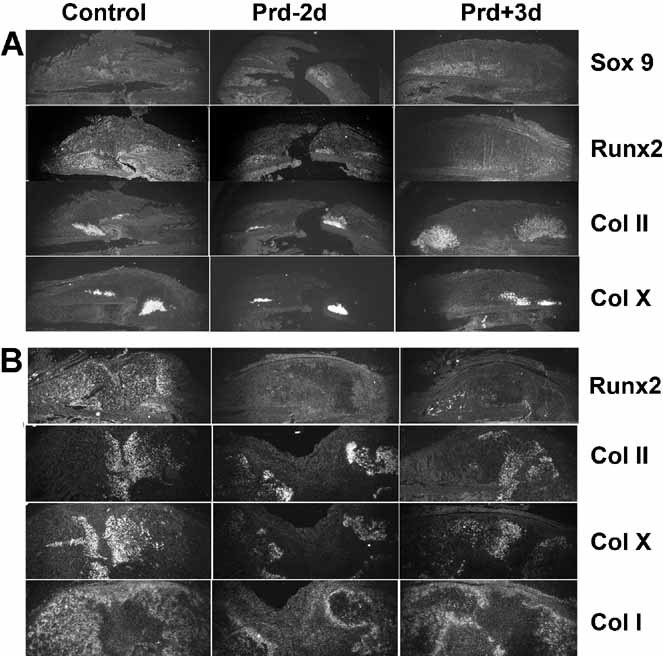
In situ hybridization analyses. Sections from the calluses of control mice and mice on the PRD were subjected to in situ hybridization analyses for the mRNAs indicated on the right. Data in *A* were obtained 7 days after fracture, whereas those in *B* was obtained 14 days after fracture. The upper part of each image represents half of the callus, with the original cortical bone on the lower aspect. Data represent those obtained from two sections from each of three mice for each time point and dietary condition.

In situ hybridization analyses performed 7 days after fracture demonstrated prominent areas of cells expressing *Sox9* and *ColII* in both phosphate-restricted groups. In addition, there was a decrease in the domain of cells expressing *Runx2* and *ColX*; these domains colocalized in the callus, implicating the hypertrophic chondrocytes as the source of *Runx2* (4*A*). By day 14 after fracture, the callus of the mice fed the control diet largely was composed of *ColI*-expressing cells. There was a smaller domain of *ColX*- and *ColII*-expressing chondrocytes relative to the extensive *ColI* domain in the control callus. The domain of *Runx2* expression overlapped the *ColI*- and *ColX*-expressing domains (see Fig. 4B).

When compared with the control callus at 14 days, the calluses of the mice on the PRD demonstrated a dramatic decrease in *Runx2* expression. This was associated with a decrease in *ColX*- and *ColI*-expressing cells, suggesting that phosphate restriction impairs differentiation (see 4*B*).

Quantitative RT-PCR analyses of early markers of differentiation demonstrated that the expression of *Sox9* was unaffected by phosphate restriction 5 days after fracture and was mildly decreased 10 days aftert fracture. Notably, the expression of *Sox5* and *ColII*, markers of early commitment to the chondrogenic lineage, also was increased in the phosphate-restricted mice 5 days after fracture. Peak expression levels of *aggrecan* and *MMP13* were lower in both phosphate-restricted groups compared with mice fed the control diet (5*A*). In addition, the phosphate-restricted mice failed to show the increase in *osteocalcin* mRNA expression observed in the control mice at 14 days after fracture. To define the molecular basis for impaired differentiation of cells committed to the chondrocyte lineage, the expression of transcription factors that play a key role in endochondral bone formation was examined. As demonstrated in 5*A*, the expression of *Runx3*, *Runx2*, and *osterix* was unaffected by the dietary regimen 5 days after fracture. However, by 10 days after fracture, there was a dramatic increase in the expression of *Runx3* in the control callus, whereas expression of this transcription factor in the calluses of the mice on the PRD was not significantly increased. At 14 days after fracture, the PRD calluses failed to show the marked enhancement in the expression of *Runx2* and *osterix* mRNA observed in the control callus (see 5*A*).

**Fig. 5:**
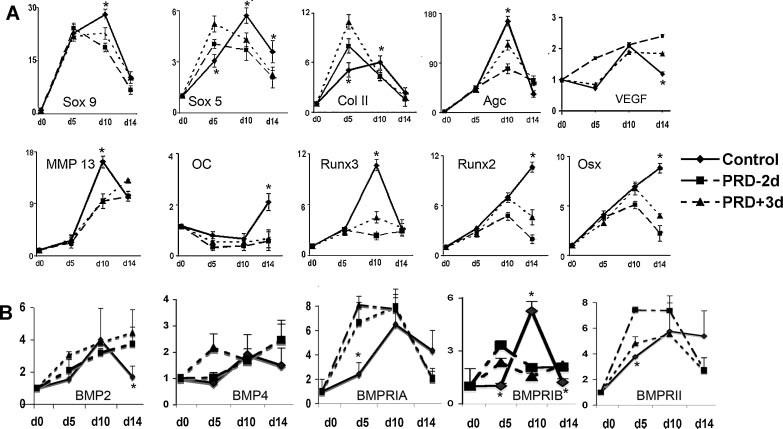
Quantitative RT-PCR analysis (*A*) Expression of mRNAs encoding markers and transcriptional regulators of chondrogenic and osteogenic differentiation was compared with that obtained from the adjacent unfractured bone. (*B*) Expression of mRNAs encoding BMP-2 and -4 and their receptors was compared with that obtained from the adjacent unfractured bone. mRNA levels from three calluses were compared with those from unfractured bones of mice fed the same diets. All data are normalized to β-actin levels in the same sample and presented as the fold change in expression relative to unfractured bone (d0). Data represents the mean ± S.D.

Since these three transcription factors are induced by BMPs,(20–22) investigations were undertaken to determine if BMP signaling was impaired in the calluses of the mice on the PRD. The expression of *BMP2* and *BMP4* was not significantly altered by phosphate restriction at 5 and 10 days after fracture (see 5*B*). Expression of *BMPRIA*, *BMPRIB*, and *BMPRII* was significantly increased in the calluses of the phosphate-restricted mice 5 days after fracture. This was accompanied by a marked reduction in the presence of nuclear pSmad-positive cells in the stromal and cartilaginous components of the fracture callus of the phosphate-restricted mice relative to that of the control mice, suggesting impaired BMP action (6*B, C* versus 6*A*).

**Fig. 6:**
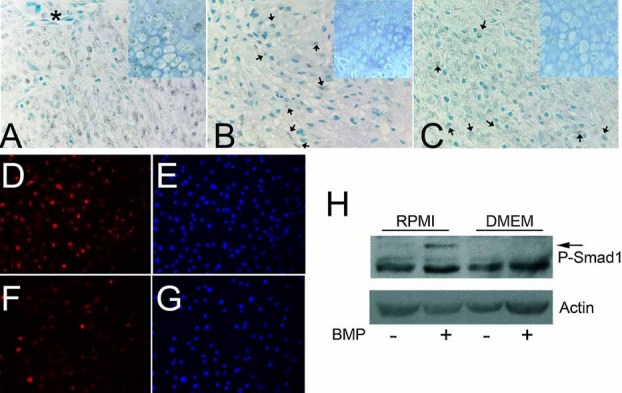
Phosphate restriction attenuates nuclear localization of pSmad1. (*A*) Control callus. Most stromal cells are positive for nuclear pSmad1 (*brown stain*); asterisk indicates a blood vessel with no nuclear pSmad in endothelial cells. (*B*, *C)* Calluses of mice placed on the PRD 2 days before or 3 days after fracture. Arrows point to the rare cells with nuclear pSmad1. Insets in the right upper corner represent chondrocytes from the same calluses. (*D–H*) ST2 cells were cultured in RPMI (5.6 mM phosphate; *D*, *E*) or DMEM (1 mM phosphate; *F*, *G*). Immunocytochemistry for pSmad was performed 90 minutes after rhBMP-2 (*D*, *F*). DAPI staining was performed to permit identification of nuclei (*E*, *G*). (*H*) Phosphate restriction attenuates Smad phosphorylation. Cells were cultured as earlier, and lysates were subjected to Western blot analyses for pSmad (*arrow*). Data are representative of those obtained in three independent experiments.

To confirm that activation of Smad1 is impaired by low phosphate in stromal cells, ST2 cells were maintained in RPMI/10% FBS or DMEM/10% FBS. As shown in 6*D*, 90 minutes after BMP-2 treatment, nuclear pSmad signals predominated in the cells cultured in RPMI, which contains 5.6 mM phosphate, and were rare in the cells cultured in DMEM (see 6*F*), which contains 1 mM phosphate. Western blot analyses demonstrate impaired Smad phosphorylation in response to BMP-2 in the cells cultured in DMEM (see 6*H*).

**Fig. 7:**
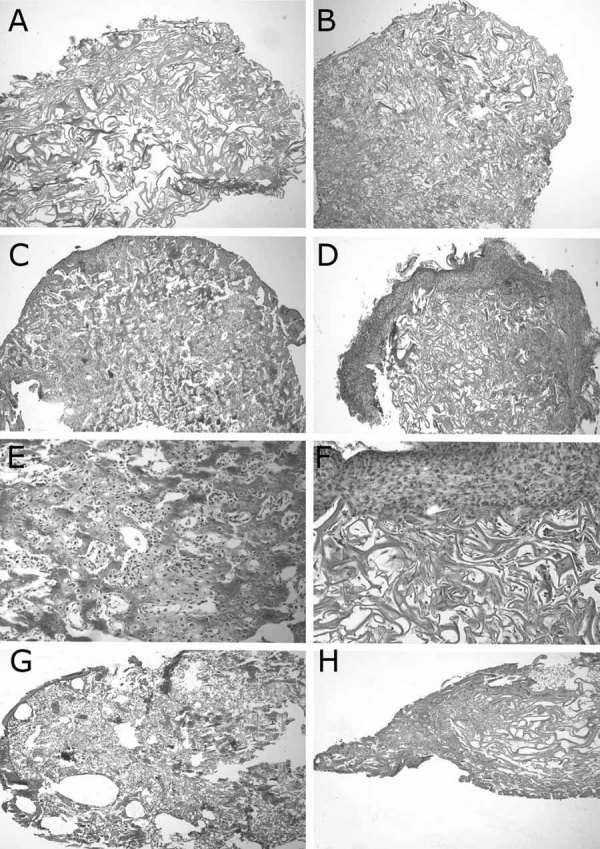
Phosphate restriction impairs rhBMP-2-induced ectopic bone formation. BFAs were performed in mice fed chow (*A*, *C*, *E*, *G*) or the PRD (*B*, *D*, *F*, *H*) after implantation of a rhBMP-2-impregnated Helistat sponge. Histology was evaluated 6 (*A*, *B*), 9 (*C*. *D*; high-power E&F), and 14 (*G*) days after implantation. Sections are representative of those obtained in five independent experiments for each time point.

To confirm that hypophosphatemia impairs BMP action in vivo, ectopic bone-formation assays (BFAs) were performed by implanting BMP-2-impregnated Helistat sponges subcutaneously into C57Bl/6J mice, which then were placed on the PRD or control diet. Six days after BFA, there was little invasion of cells into the sponge in either group of mice (7*A, B*). However, by 9 days after BFA, the sponges in the mice on the control diet exhibited a high degree of cellularity, with evidence of mesenchymal cell differentiation into chondrocytes (see Fig. 7*C, E*). In contrast, in the mice fed the PRD, there was marked hypocellularity, with cells occupying only the rim of the sponge (see Fig. 7*D, F*). By 14 days, when the sponges in the control mice were replaced by bone and a marrow cavity, the sponges from the mice subjected to the PRD showed no further increase in cellularity (see Fig. 7*G, H*).

**Fig. 8:**
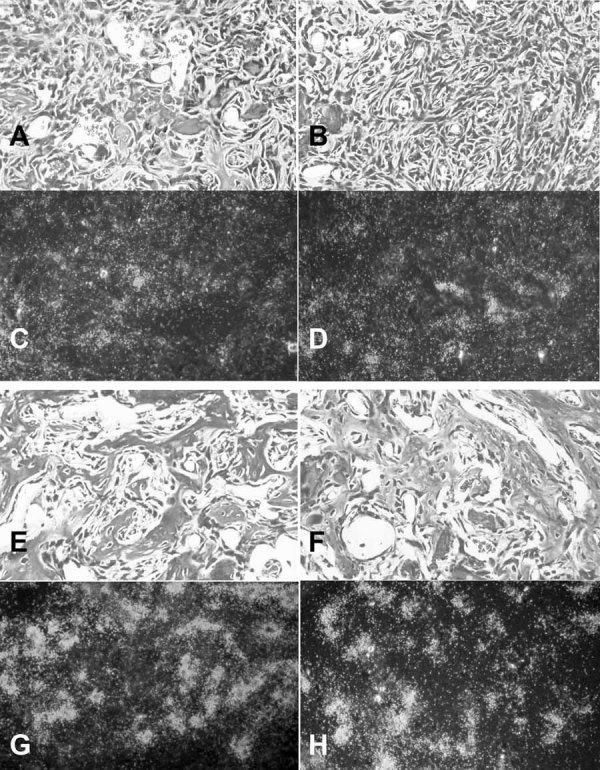
Intramembranous bone formation. Marrow ablation was performed on mice fed a control diet (*A*, *C*, *E*, *G*) or phosphate-restricted diet 2 days prior to the procedure (*B*, *D*, *F*, *H*). Stromal cell response and differentiation were evaluated 5 (*A*, *B*, *C*, D) and 9 days after marrow ablation (*E*, *F*, *G*, *H*). In situ hybridization for *osteocalcin* mRNA (*C*, *D*, *G*, *H*) was performed to evaluate stromal cell differentiation.

Both the ectopic BFA and long bone fractures recapitulate embryonic endochondral bone formation. In order to address whether hypophosphatemia impairs intramembranous bone formation as well, marrow ablation studies (a model of injury-induced bone repair after surgical reaming) were performed in mice placed on the PRD 2 days prior to the procedure. Phosphate restriction did not impair the stromal cell response either at 5 or 9 days after marrow ablation (Fig. 8*B, F*) compared with that observed in mice on the control diet (see Fig. 8*A, E*). Based on in situ hybridization for *osteocalcin* expression, differentiation of these cells into osteoblasts was not adversely affected by phosphate restriction (Fig. 8*D, H* versus 8*C*, *G*).

## Discussion

The skeletal abnormalities seen in hypophosphatemic disorders highlight the importance of phosphate in growth plate maturation and skeletal mineralization.(14,23–25) However, little is known about the role of phosphate in skeletal development and repair. The current investigations identify a critical role for phosphate in fracture repair and implicate a role for phosphate in normal skeletal development and responsiveness to BMPs.

The BMPs belong to the transforming growth factor β superfamily of growth factors. When implanted subcutaneously, BMP-2 leads to ectopic bone formation by a series of events that recapitulate embryonic endochondral bone formation.(26) Furthermore, BMPs have been used to promote healing of fractures, including those that exhibit nonunion, a pathologic condition associated with failure of fracture healing.(27–30) BMPs also have been shown to have critical roles in the development of other organ systems.(31,32) as well as in limb patterning.(33) Misexpression of the BMP inhibitor noggin in chick limb buds prevents both mesenchymal condensation and chondrogenic differentiation of these mesenchymal cells.(34,35) Although BMP2, BMP4, and BMP7 are expressed throughout limb development, their functions are not completely redundant. In the absence of BMP2 and BMP4, chondrogenesis proceeds normally, with the exception of a delay in hypertrophic differentiation, but osteogenesis is markedly impaired.(33) Absence of BMP14 delays fracture healing,(36) whereas ablation of BMP4 has no effect.(37) In contrast, mice lacking BMP2 in their limbs exhibit a decrease in bone mineral density, develop spontaneous fractures, and display markedly impaired fracture healing.(38) The dramatic fracture phenotype observed in these mice, despite normal levels of BMP4 and BMP7 mRNA, suggests that BMP2 is specifically required for the earliest stages of fracture repair. Absence of BMP2 may impair the inflammatory response to fracture or, alternatively, deplete the pool (or impair the function) of precursor cells that are normally recruited to the fracture site during the inflammatory phase. The latter explanation would be most consistent with our investigations in the hypophosphatemic fracture model: The acute nature of the phosphate restriction in these studies is unlikely to affect the pool of precursor cells significantly.

The most striking pathologic abnormality in the hypophosphatemic calluses is the marked impairment in progression of chondrogenic differentiation of the mesenchymal cells that are recruited to the fracture callus, analogous to that observed with postnatal impairment of BMP2 signaling.(39) The recruitment of mesenchymal cells, which occurs during the initial inflammatory phase of fracture repair, is thought to depend on growth factors, cytokines, and prostaglandins, but studies in the BMP2 null mice demonstrate a role for this growth factor in this process. Despite the BMP2 resistance we observed, and in contrast to mice lacking BMP2 in their limbs, the recruitment of cells during the inflammatory phase of fracture repair was not profoundly impaired by hypophosphatemia. This suggests that the duration or degree of impaired BMP2 function is an important determinant of this phase of fracture repair. Regardless of whether the mice are normophosphatemic (PRD+3) or hypophosphatemic (PRD–2) at the time of fracture, the molecular and histologic phenotype of the fracture callus is the same. This suggests that the recruitment of mesenchymal cells to the fracture site and commitment to the chondrogenic lineage are not affected by hypophosphatemia because the PRD–2d mice are hypophosphatemic during these stages, whereas the PRD+3d mice are not. However, the subsequent differentiation of these cells is severely impaired. This impaired differentiation leads to a decrease in callus volume primarily owing to the fact that there is a marked reduction in chondrocytes and osteoblasts resulting in an increase in cell density owing to the presence of less matrix. A minor reduction in stromal cell proliferation also contributes to the decrease in callus size. The reduction in callus matrix is also likely to be responsible for the reduction in mineralized tissue volume (MTV/TV) observed. Interestingly, despite the presence of hypophosphatemia, the density of the mineral that is present in the callus (CMD) is only mildly reduced.

Because of the effects of BMP-2 on stromal cell differentiation and the essential requirement of this growth factor for fracture repair, we examined the effects of low phosphate on BMP2 signaling in the callus, in cultured stromal cells, and in ectopic BFAs. The impaired Smad phosphorylation observed in vivo and in vitro in the stromal cells and striking paucity of mesenchymal cell invasion observed in the ectopic BFAs performed in hypophosphatemic mice demonstrate that BMP2 signaling in these cells is impaired under low-phosphate conditions both in vitro and in vivo. Similarly, there was a dramatic decrease in the presence of pSmad-positive chondrocytes in the calluses of the mice on the PRD. Whether this effect of phosphate restriction on *BMP2* signaling is limited to cells involved in endochondral bone formation is currently unknown. Investigations in HaCaT (human keratinocyte) cells and in HeLa cells (from a human cervical cancer) demonstrate rigorous Smad phosphorylation in response to BMPs despite the fact that they are cultured in DMEM (1 mM phosphate), providing the rationale for choice of this culture medium in our studies.(40,41) Similarly, primary bovine articular chondrocytes cultured in DMEM/F12 (1 mM phosphate) demonstrate nuclear pSmad in response to BMP treatment.(42) Interestingly, the observation that hypophosphatemia does not significantly alter the differentiation of stromal cells in the marrow ablation studies, which recapitulate intramembranous bone formation, suggests that the actions of BMPs are not essential in this model or that the stromal cell population that reconstitutes bone in this model differs from that recruited in the fracture model and ectopic bone formation assays, which recapitulate endochondral bone formation. Notable in this respect is the observation that periosteal injuries heal by endochondral bone formation, whereas the endosteal injuries heal by intramembranous bone formation.(43)

The results of the ectopic BFAs and investigations in ST2 cells, combined with those of the fracture studies, suggest that inflammation-mediated cell recruitment is BMP-independent. However, BMP-2 may be required to sustain the pool of precursor cells that are recruited to the fracture site or to maintain their functional activity. The initiation of hypophosphatemia just prior to or after the fracture in our model would circumvent this potential impairment of BMP2 action on progenitor cells, thus leading to a less severe phenotype than that observed in the mice where BMP2 is ablated from the limbs during embryonic development. Nonetheless, our data point to a critical role for phosphate in fracture healing and endochondral bone repair. They also suggest that BMP resistance has a pathophysiologic role in the etiology of the impaired fracture repair observed in hypophosphatemic states.
